# What proportion of calories given to critically ill patients come from propofol?

**DOI:** 10.1186/2197-425X-3-S1-A188

**Published:** 2015-10-01

**Authors:** AKA Abi Musa Asa'ari, ST Passey, M Mendis, B Carr

**Affiliations:** Critical Care Unit, University Hospitals of North Midlands Trust, Stoke-on-Trent, United Kingdom

## Introduction

Establishing nutrition support is a priority in the management of the acutely unwell patient. Administration of proprofol by infusion is commonly used for sedation. This can significantly contribute to the total calorific input due to the lipid content. We investigated the total calorific input received in a cohort of patients in the first 7 days of nutrition support during their critical care stay.

## Objectives

We examined the current practice of our intensive care unit (ICU) looking at the total calorific input including the proportion of calories contributed from profolol infusions.

## Methods

We collected retrospective data from 50 consecutive ICU patients from July to November 2014. We included patients that received ≥7 days of enteral/parenteral feeding. Data collected included; patient demographics, type and amount of feed given and total propofol administered in the same period. We then calculated the proportion of calories from propofol (1.0kcals/ml) given to the 50 patients.

## Results

In the 50 patients established on feed,10% of the 7 day total calorific input came from propofol.

One patient (patient 2) received the highest calories from profolol at 4169 kcals.

Patient 16 had the highest proportion of calories from propofol at 33%.

## Conclusions

Use of propofol for sedation makes a significant contribution towards a patient's energy requirements. Although in situations such as traumatic brain injury (patient 2) deeper sedation may be required, care is still required over both prescription of propofol, and nutrition support to take account of the additional calories provided as Intralipid with the propofol.

**Figure 1:**
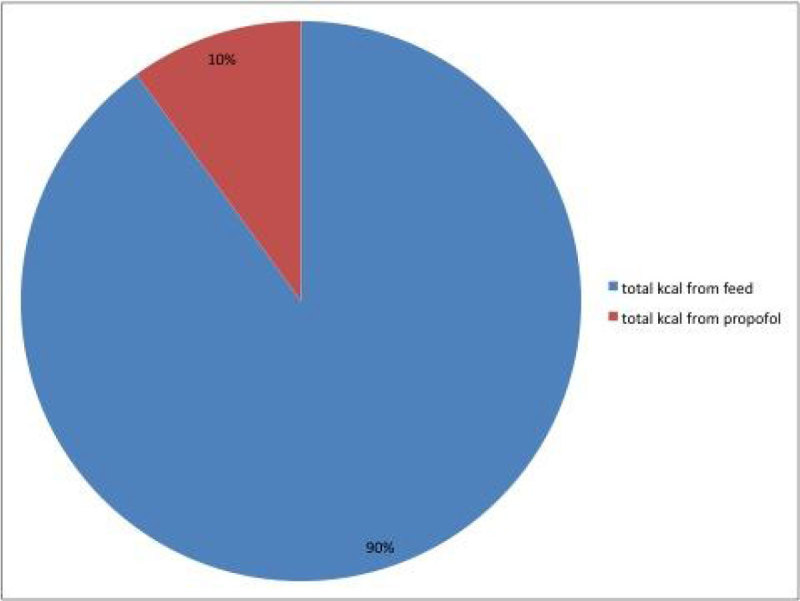
**Percentage of calories from feed and propofol.One patient (patient 2) received the highest calories from profolol at 4169 kcals.**

**Figure 2:**
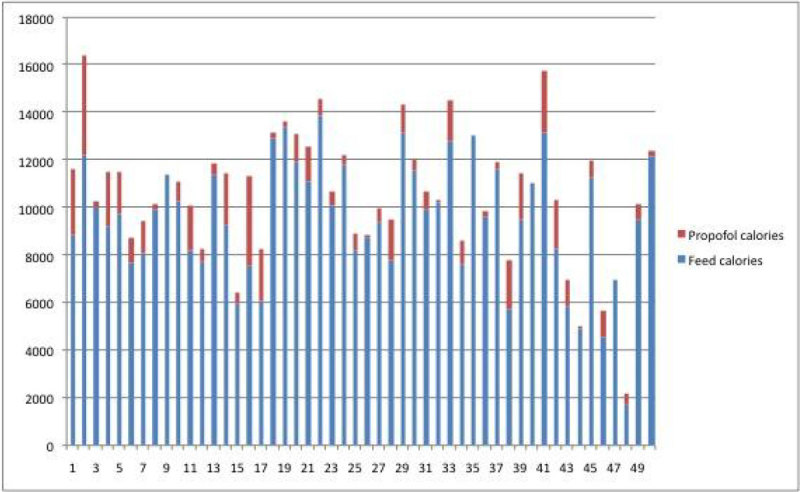
**Calories from feed/propofol in individual patients.Patient 16 had the highest proportion of calories from propofol at 33%.**

**Figure 3:**
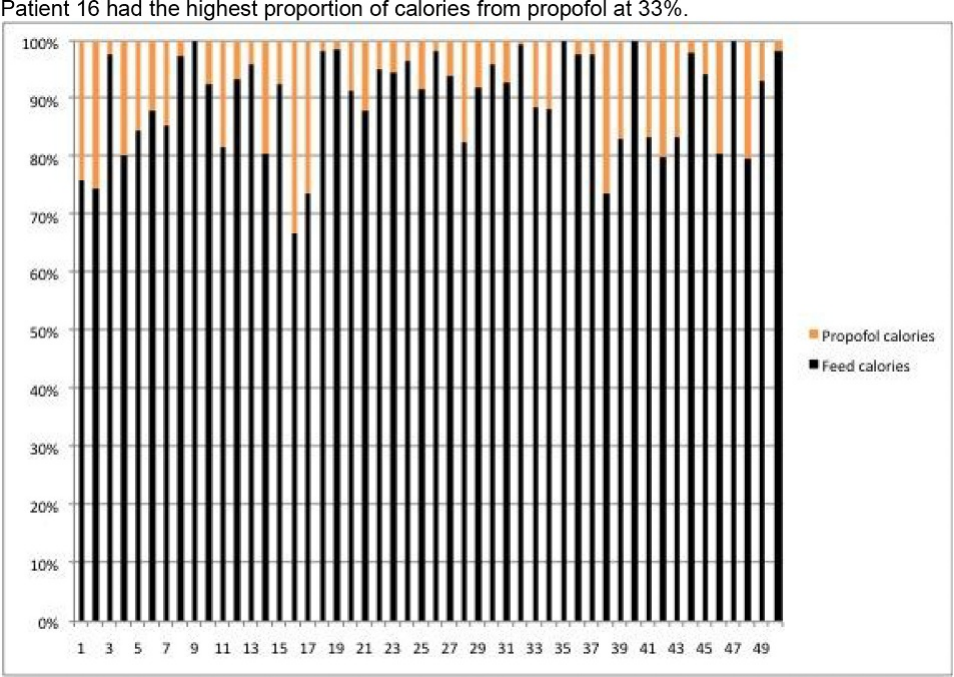
**Calories from feed/propofol in percentages.**

